# The factor influencing the rate of ROSC for nontraumatic OHCA in New Taipei city

**DOI:** 10.1097/MD.0000000000028346

**Published:** 2021-12-30

**Authors:** Yi-Chung Yu, Chin-Wang Hsu, Shih-Chang Hsu, Jin-Lin Chang, Yuan-Pin Hsu, Shih-Min Lin, Ying-Kuo Liu

**Affiliations:** a Emergency Department, Department of Emergency and Critical Medicine, Wan Fang Hospital, Taipei Medical University, Taipei, Taiwan; b Department of Emergency Medicine, School of Medicine, College of Medicine, Taipei Medical University, Taipei, Taiwan; c Fire Department, New Taipei City Government, New Taipei City, Taiwan; d Emergency Department, Camillian Saint Mary's Hospital Luodong, Yi-Lan, Taiwan.

**Keywords:** cardiopulmonary resuscitation interruption, chest compression fraction, out-of-hospital cardiac arrest, patient transportation, prehospital care

## Abstract

Return of spontaneous circulation (ROSC) from out-of-hospital cardiac arrest (OHCA) is critical for the Emergency Medical Services System. When compared to other developed countries, Taiwan has lower rate of ROSC in OHCA patients.

We conducted a retrospective study of cardiac arrest using The Emergency Medical Service Dispatching Center in Northern Taiwan and The Prehospital Care System of New Taipei City Paramedic Service. Patients suffering from nontraumatic OHCA between August of 2019 to February of 2020 were included. We analyzed the cardiopulmonary resuscitation (CPR) quality parameters such as chest compression interruptions, bystander CPR, shockable rhythm, CPR interruption, chest compression fraction (CCF) average, patient transportation in buildings, and adrenaline injection during CPR. Multivariable logistic regression analysis was performed to assess the relationship between potential independent variables and ROSC.

In our study, we involved 1265 subjects suffering from nontraumatic OHCA, among which 587 patients met inclusion criteria. We identified that CCF> 0.8, chest compression interruption greater than 3 times, and patient transportation in the building were the most critical factors influencing ROSC. However, patient transportation in a building was identified as a dependent predictor variable (*P* = .4752).

We concluded that CCF > 0.8 and chest compression interruption greater than 3 times were essential factors affecting the CPR ROSC rate. The most significant reason for suboptimal CCF and CPR interruption is patient transportation in a building. Improving the latter point could facilitate high-quality CPR.

## 1 Introduction

Return of spontaneous circulation (ROSC) from out-of-hospital cardiac arrest (OHCA) represents an ongoing challenge to emergency medical services (EMS) systems among many countries. According to North America's Prehospital Care Response System Report, 33.2% of nontraumatic OHCA patients experienced ROSC from 2011 to 2015.^[1]^ Jan-Thorsten Gräsner et al^[2]^ demonstrated that data on the percentage of ROSC for all European countries was 28.6%. The variability of OHCA prehospital ROSC rate ranged from 16% in Italy, 30% in Ireland, up to 37% in Norway.[^3^,^4^,^5^] Pan Asian Resuscitation Outcome Study clinical research network reported OHCA prehospital ROSC rate ranged from 3.4% in Malaysia to 21.0% in Thailand between 2009 and 2012.^[6]^ However, from 2011 to 2013, the ROSC rate of nontraumatic OHCA patients in rural areas in Taiwan was 19.7% and 27.7% in the Great Taipei Area.[^7^,^8^] Based on the above information, the ROSC rates are higher in developed countries than in Taiwan.

Improving survival from OHCA continues to challenge EMS systems.^[9]^ As it has been highlighted in guidelines and consensus statements published by both the American Heart Association and International Liaison Committee on Resuscitation, providing better cardiopulmonary resuscitation (CPR) quality is a critical factor in improving outcomes from OHCA.[^10^,^11^] CPR quality, including chest compression fraction (CCF), chest compression rate, chest compression depth, initial shockable rhythm, peri-shock pause, adrenaline injection, and CPR interruption more than 2 times, have all been associated with improved survival to hospital discharge from OHCA.[^12^,^13^,^14^,^15^,^16^,^17^,^18^,^19^]

The EMS Dispatching Center in Northern Taiwan and the Prehospital Care System of New Taipei City Paramedic Service provides emergency care for a population of 4.1 million people in urban and rural settings within a geographic area of 2053 km^2^. Paramedics in these regions respond to over 2000 OHCA per year. The paramedics equate with complete advanced life support skills, a small number of medications, mechanical CPR devices, and automated external defibrillators. Nevertheless, there was no quality control team to monitor New Taipei City EMS efficacy at the OHCA resuscitation mission until 2019.

New Taipei City has lower ROSC rate in OHCA patients than other countries. This study aimed to investigate OHCA events in New Taipei City regarding incidence and features. We further investigated factors that may influence high-quality CPR performance and ROSC rate. Reporting and analyzing those parameters that influence CPR quality (chest compression interruptions and patient transportation in buildings) can identify the best intervention method to improve the services, furthermore, improve the ROSCs rate in New Taipei City.

## 2 Method

We conducted a retrospective study of cardiac arrest using the data of The Emergency Medical Service Dispatching Center in Northern Taiwan and the Prehospital Care System of New Taipei City Paramedic Service. A digital video and audio-recording camera was set up in every emergency medical technician (EMT) helmet combined with data extracted from the Zoll X Series defibrillator to assess the effectiveness of CPR performance. EMTs recorded each patient's general data and resuscitation parameters (including prehospital CPR timing, equipment use, automated external defibrillator rhythm, and intervention, etc) uploaded CPR performance parameters to New Taipei City Paramedic Service databank. The exclusion criteria were: patients less than 18 years old; patients with missing data of general characteristics; patients with missing CPR data. We extracted their data and calculated the percentage of patients with ROSC and analyzed the subgroups with chest compression interruptions, bystander CPR, shockable rhythm, CPR interruption, CCF average, patient transportation in a building, and adrenaline injection during CPR. CCF is defined as the proportion of time spent providing chest compression while the patient is pulseless and in cardiac arrest. The CPR interruption is defined as CPR interruption of more than 3 times for more than 10 seconds.

The ethics committee approved this study of the Institutional Review Board of Taipei Medical University in Taipei City, Taiwan (TMU-JIRB No. 201909006).

### 2.1 The goal of the study

The primary goal was to indicate the rate of ROSC in OHCA and determine the crucial parameters that influence the ROSC rate in New Taipei City. Then, based on the results, we could seek the best solution to improve the EMTs performance in New Taipei City.

### 2.2 Statistical analysis

Continuous variables were expressed as means and Standard Deviation (SD) or medians and the interquartile range (IQR). The categorical variables were summarized by using descriptive statistics with frequencies (percentages) or count. Differences between groups were analyzed for significance by the Chi-square test. Multivariable logistic regression analyses were performed to assess the relationship between potential independent variables and ROSC. Variables significant in Chi-square analysis were included in the multiple logistic regression analysis to identify independent predictors. Statistical tests were 2-sided, and *P* values less than .05 were considered statistically significant. Statistical analysis of the study's data was made using R 3.2.4 software (R Foundation for Statistical Computing, Vienna, Austria).

## 3 Result

**Figure 1:**
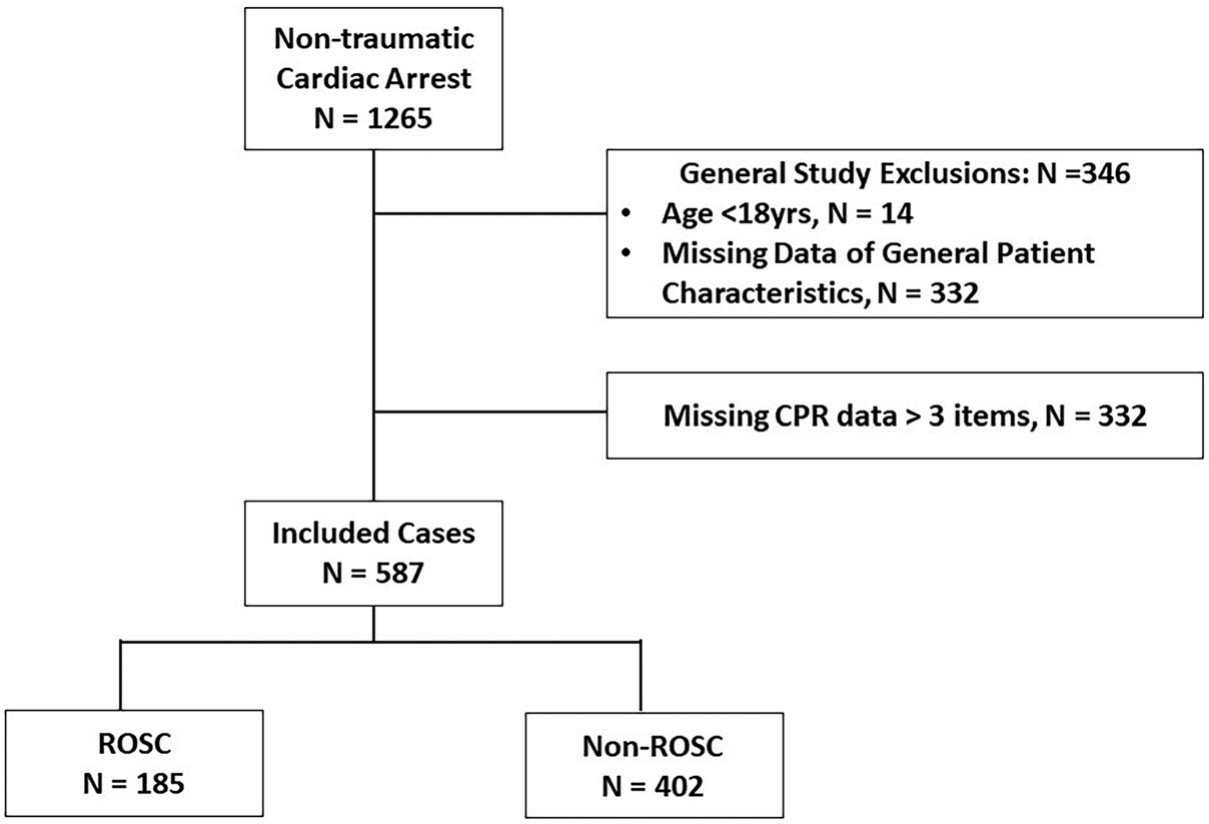
Study scheme.

Data from 1265 individuals were collected from The Emergency Medical Service Dispatching Center in Northern Taiwan and the Prehospital Care System of New Taipei City Paramedic Service. We excluded 678 patients due to one of the following selection criteria: age < 18 years old (n = 14), missing data of general patient's characteristic (n = 332), or mission CPR data (n = 332) (Fig. 1).

**Figure 2:**
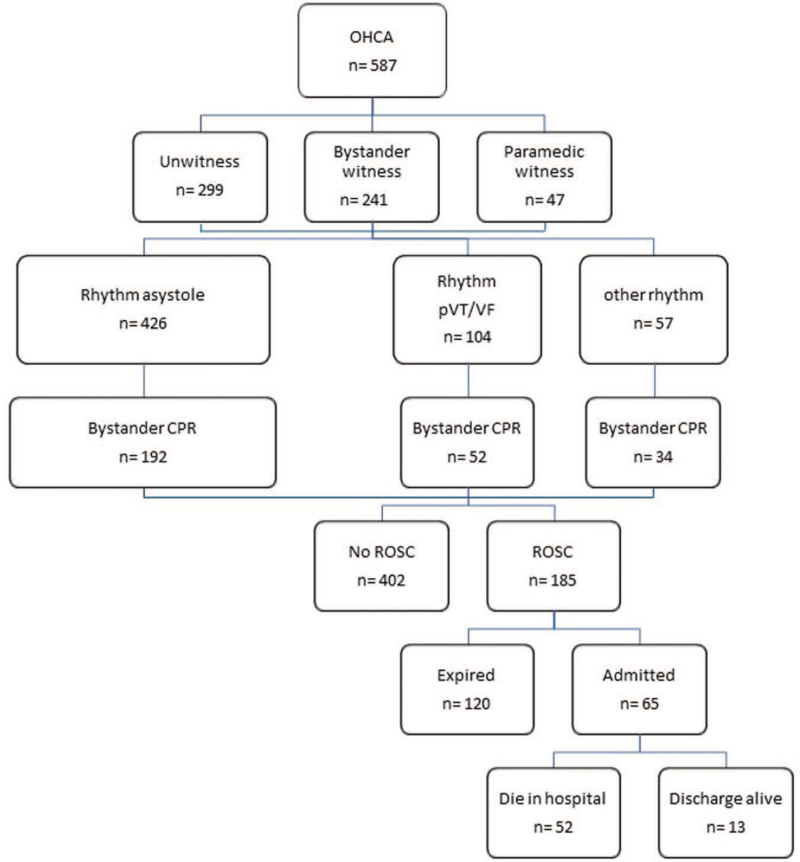
The Utstein template.

Results are also summarized according to the Utstein style template (Fig. 2).

Our analysis includes 587 patients presenting with OHCA from the selected study sites. There were 211 women and 376 men with a mean age of 65.3 ± 16.9 years. Two hundred eighty-eight out of 587 (49%) patients had a witnessed arrest, of which only 16% (47 out of 88) of cases were witnessed by the paramedic. Overall, 185 patients had ROSC, of which 35% (65 out of 185) survived to be admitted to hospital. When these survivors were followed till the end of hospital stay, only 20% (13 out of 65) of patients survived and were later discharged. Most of the patients having a ROSC died within a few hours of the event and the likely cause of death was hemodynamic instability rather than neurologic sequelae. Upon the arrival of emergency service personnel, shockable rhythm can be detected in 17.7% (104 out of 587) of OHCA patients, of which 50% (52 out of 104) of cases received bystander CPR. Asystole rhythm was the most common form of presentation and could be detected in 72.5% OHCA patients (426 out of 587), of which 45% (192 out of 426) of cases received bystander CPR.

Ultimately, 587 prehospital OHCA patients were selected for further analysis, in which 185 cases experienced ROSC (31.51%). Patient baseline characteristics for the sample population are described in Table 1. The mean age of the study population was 65.3 years old (standard deviation: 16.97, IQR: 56-79), and 376 (64.05%) cases were male. The most common comorbidities were a history of hypertension (32.20%), diabetes mellitus (25.21%), heart disease (21.47%), and malignancy (8.01%).

**Table 1:** Patient baseline characteristics.

	All patients	Patients with ROSC	Patients without ROSC	
	n = 587	n = 185	n = 402	*P* value
Age: mean (SD)	65.3 (16.97)	64 (15.7)	66.81 (17.47)	.1406
Male: n (%)	376 (64.05)	125 (67.57)	251 (62.44)	.3546
Comorbidities				
Heart disease: n (%)	126 (21.47)	40 (21.62)	86 (21.39)	.9272
Diabetes mellitus: n (%)	148 (25.21)	43 (23.24)	105 (26.12)	.3979
Hypertension: n (%)	189 (32.20)	64 (34.59)	125 (31.09)	.3877
Malignancy: n (%)	47 (8.01)	18 (9.73)	29 (7.21)	.1105

In our study, 47.36% of the subjects received bystander CPR, and the initial shockable rhythms were 17.72%. The mean CPR duration time and interruption duration time were 1275 seconds and 331.5 seconds, respectively. The mean CCF were 0.74 (IQR 0.54-0.85) and CCF > 0.8 was 40.37%. Epinephrine injection was found in 39.01% of OHCA patients. Additional details of the patients’ CPR characteristics are presented in Table 2.

**Table 2:** Patient demographics.

	All patients	Patients with ROSC	Patients without ROSC
	n = 587	n = 185	n = 402
Bystander CPR: n (%)	278 (47.36)	85 (45.95)	193 (48.01)
Shockable rhythm: n (%)	104 (17.72)	42 (22.70)	62 (15.42)
CPR duration: s	1275	1250	1289
interruption duration: s	331.5	212	425
CCF average: median (IQR)	0.74 (0.85-0.54)	0.83 (0.89-0.71)	0.67 (0.81-0.51)
CCF > 80%: n (%)	237 (40.37)	123 (66.49)	114 (28.36)
Epinephrine: n (%)	229 (39.01)	65 (35.14)	164 (40.80)

This study identified that CCF > 0.8, chest compression interruption greater than 3 times, and patient transportation in the building were the most critical factors influencing ROSC (Table 3). We further employed multivariate analysis to verify the independent predictors of ROSC, the CCF (*P* < 1 ×10-10), chest compression interruption (*P* < .001), and patient transportation in a building (*P* = .4752). In contrast, patient transportation in a building was a dependent predictor variable (Table 4).

**Table 3:** Characteristics of patients.

	Non-ROSC	ROSC	*P* value
Age			
≧65	223	92	.2273
<65	179	93	
Sex			
Male	252	124	.3546
Female	150	61	
CCF			
≧80%	113	124	<.00001
<80%	289	61	
Bystander CPR			
Yes	192	86	.7598
No	206	99	
Shockable rhythms			
Yes	59	29	.7902
No	345	154	
Epinephrine			
Yes	163	66	.1827
No	218	115	
Chest compression interruption			
≧3	139	24	<.00001
<3	263	161	
Patient transportation in building			
Yes	207	69	<.0019
No	195	116	

**Table 4:** Logistic regression analysis.

	OR	95% CI	*P* value
CCF	3.654	2.485-5.431	< 1 ××10^−10^
Chest compression interruption	0.406	0.237-0.675	<.001
Patient transportation in building	0.864	0.578-1.290	.4752

## 4 Discussion

**Figure 3:**
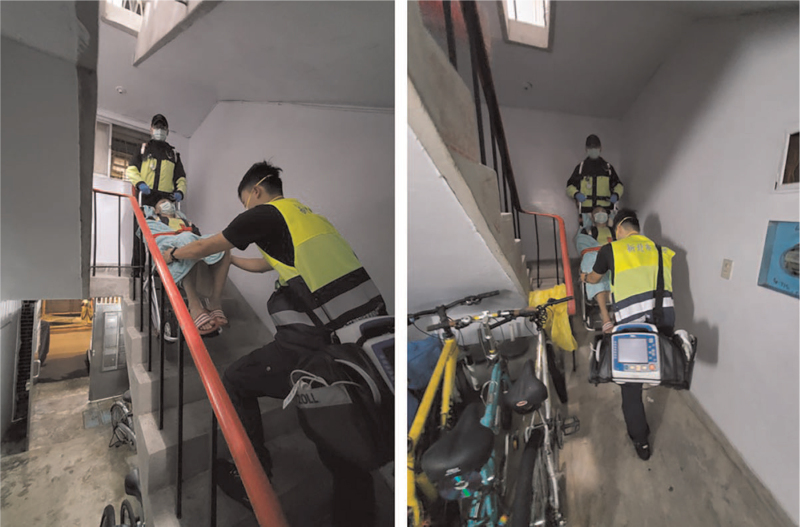
Patient transportation in Taipei Metropolitan areas.

In this study, 185 out of 587 subjects experienced prehospital ROSC (31.51%) in New Taipei City during our study period. For CPR quality analysis, we further analyzed several factors affecting CPR quality. In our data set, the crucial factors influencing the ROSC rate were CCF > 0.8, chest compression interruption greater than 3 times, and patient transportation in the building. Furthermore, we identified that patient transportation in a building is not an independent factor affecting the CPR ROSC rate. In New Taipei City, it is common for EMT to transport OHCA patients in small apartment buildings, which results in poor CPR performance. These small apartments usually have no elevators, and OHCA victims could only be transported via extremely narrow stairs where the mechanical chest compression system (ex: LUCAS or autopulse) hardly worked. As a result, EMTs could only perform manual chest compression intermittently while reaching the stairway platform, which caused frequent CPR interruption (Fig. 3).

The study from Canada reported that the median CCF was 0.89^[20]^; however, in our study, the median of CCF was only 0.74. Moreover, the mean CPR Interruption duration was 222 seconds in Denmark,^[21]^ whereas our CPR mean interruption duration was 331.5 seconds. Based on our results, we believe that patient transportation in the building may substantially influence CCF and chest compression interruption in New Taipei City.

According to previous research, a higher post-OHCA survival rate is more likely in patients who received bystander CPR,^[22]^ which contrasts with our result. The explanation is that compared with North America and Europe, the popularization of bystander CPR has been relatively delayed in Asia; thus, bystander CPR's quality might be lower in this region. In addition, 1 study showed that the threshold of EMS protocols for initiating resuscitation is lower in Asian countries,^[23]^ which was likely to contribute to the difference in the survival rates. Therefore, improved BLS education for laypeople to obtain the skills necessary to recognize OHCA and early CPR with ongoing medical dispatcher training would provide better dispatcher-assisted CPR and ensure high-quality bystander CPR.

Our data demonstrated that shockable rhythm does not affect the survival rate. We further surveyed the EMS resuscitation effect by analyzing the video recorder and noticed that some EMT did not stop chest compression while automated external defibrillator analyzed rhythm, leading to possible false interpretation as shockable rhythm and then conducting ineffective DC shock. Furthermore, AL Hallstrom et al^[24]^ observed that the ROSC rate had no significant difference between OHCA patients initially found in PEA or Asystole, subsequently received defibrillation and OHCA patients remained in a non-shockable rhythm, they concluded that for initially non-shockable rhythm patients, ALS providers should focus less on the defibrillator and more on other treatment methods such as high-quality CPR with minimal interruption, appropriated ventilation, and identification and treatment of reversible causes.

Administration of epinephrine seems not to affect the ROSC rate in our study group. One previous study indicated that repeated doses of adrenaline are associated with decreasing chances of survival.^[25]^ Attaran^[26]^ 2010 pointed out that epinephrine could be either a curse or a cure in cardiac arrest. Moreover, its value depends on the dosage, the administration's timing, and the cardiac arrest's initiating factor. Nevertheless, earlier administration of epinephrine is not the cure by itself. Its effect on OHCA patients should be integrated into a series of essential steps, including cardio cerebral resuscitation and post-resuscitation care.

Our results confirmed a trend of poor survival rate of frequent chest compression interruption and low CCF ratio, which is an independent predictor variable indicated by statistical analysis. Additionally, our study suggests that patient transportation in a building is a dependent predictor variable of chest compression interruption and CCF. Therefore, we believe that modifying our current method of patient transportation in a building could increase CCF and minimize chest compression interruption.

We currently suggest that EMS should introduce the evacuation chair plus Mechanical CPR to facilitate CPR performance and minimize chest compression interruption during patient transportation, especially when EMTs have to resuscitate OHCA patients in a limited working space environment.

New Taipei city belongs to Taipei metropolitan area, thus the data can apply to other metropolitan cities in Taiwan. We pointed out the unique problem of patient transportation impact on chest compression quality which eventually influences resuscitation outcome. This finding can apply to other Asia metropolitan areas with a crowded living environment and many old buildings interfering resuscitation mission such as New Taipei City. We hope that our study's findings can be helpful to other areas that face similar scenario and remind them to re-evaluate their resuscitation mission, facilitate CPR performance, and improve OHCA patient outcomes.

### 4.1 Limitation of the study

Firstly, although we provided staff training in reporting sheet completion at the designated emergency departments and EMSs, some data were missing. We believe that it may influence the overall OHCA data interpretation. Secondly, we also attempted and failed to identify possible initial cardiac collapse cases at the beginning of the study. Therefore, this variable was removed from our analysis. We set up a quality control team to monitor and evaluate New Taipei City EMS efficacy at the OHCA resuscitation mission since August 2019. The further investigation will focus on EMS OHCA data quality control and the CPR quality evaluation. Finally, the lack of long-term outcomes and the small number of subjects weakens the evidence in this study. Because the determination of the 12-month survival status and quality of life has been emphasized in the newly revised Utstein survey,^[27]^ we have started to process this modification to the registry system to include these variables in future studies.

## 5 Conclusion

We concluded that CCF > 0.8 and chest compression interruption greater than 3 times were essential factors affecting the ROSC rate. The most significant reasons for suboptimal CCF and CPR interruption are patient transportation in a building. This study determines that minimizing patient transportation in a building may facilitate high-quality CPR.

## Acknowledgments

We appreciate the excellent performance of EMTs and the quality assurance of the emergency medical service dispatching center in northern Taiwan and the prehospital care system of the New Taipei City Paramedic Service. Their commitment and accomplishments improved prehospital care.

## Author contributions

All authors have read and agreed to published version of the manuscript.

**Conceptualization:** Y.K.L.

**Supervision:** C.W.H and S.M.L.

**Visualization:** Y.P.H.

**Writing – original draft:** Y.C.Y. and S.C.H.

**Writing – review & editing:** J.L.C.
